# Expression Profiling of *FLOWERING LOCUS T*-*Like* Gene in Alternate Bearing ‘Hass' Avocado Trees Suggests a Role for *PaFT* in Avocado Flower Induction

**DOI:** 10.1371/journal.pone.0110613

**Published:** 2014-10-17

**Authors:** Dafna Ziv, Tali Zviran, Oshrat Zezak, Alon Samach, Vered Irihimovitch

**Affiliations:** 1 Institute of Plant Sciences, The Volcani Center, Agricultural Research Organization, Bet-Dagan, Israel; 2 The Robert H. Smith Institute of Plant Sciences and Genetics in Agriculture, Faculty of Agriculture, Food and Environment, The Hebrew University of Jerusalem, Rehovot, Israel; NARO Institute of Fruit Tree Science, Japan

## Abstract

In many perennials, heavy fruit load on a shoot decreases the ability of the plant to undergo floral induction in the following spring, resulting in a pattern of crop production known as alternate bearing. Here, we studied the effects of fruit load on floral determination in ‘Hass' avocado (*Persea americana*). De-fruiting experiments initially confirmed the negative effects of fruit load on return to flowering. Next, we isolated a *FLOWERING LOCUS T-like* gene, *PaFT*, hypothesized to act as a phloem-mobile florigen signal and examined its expression profile in shoot tissues of *on* (fully loaded) and *off* (fruit-lacking) trees. Expression analyses revealed a strong peak in *PaFT* transcript levels in leaves of *off* trees from the end of October through November, followed by a return to starting levels. Moreover and concomitant with inflorescence development, only *off* buds displayed up-regulation of the floral identity transcripts *PaAP1* and *PaLFY*, with significant variation being detected from October and November, respectively. Furthermore, a parallel microscopic study of *off* apical buds revealed the presence of secondary inflorescence axis structures that only appeared towards the end of November. Finally, ectopic expression of *PaFT* in *Arabidopsis* resulted in early flowering transition. Together, our data suggests a link between increased *PaFT* expression observed during late autumn and avocado flower induction. Furthermore, our results also imply that, as in the case of other crop trees, fruit-load might affect flowering by repressing the expression of *PaFT* in the leaves. Possible mechanism(s) by which fruit crop might repress *PaFT* expression, are discussed.

## Introduction

Most crop trees produce fruits in a biennial pattern of crop production known as alternate bearing (AB) in which a heavy crop-load, or *on* year, is followed by a low crop load, or *off* year [1]. Based on studies performed in various fleshy fruit species, the emerging concept explaining this phenomenon is that AB results from developing fruits competing with the vegetative shoot apex for resources required for growth. As a consequence, trees bearing a heavy crop load are typically characterized by reduced vegetative growth. A lack of new nodes and/or a reduction in the ratio of nodes that will undergo flower transition causes a significant reduction in flowering production the following season. Furthermore, developing fruit, or the memory of high fruit load, also represses return flowering, again negatively influencing yield the next season (see reviewed by [1]–[3]).

The ‘Hass' avocado (*Persea americana* Mill.) cultivar dominates the global avocado industry. Yet, despite its popularity, this cultivar is known to be problematic due to its tendency towards biennial flowering and fruit production [4]. Typically, in the growing region of the northern hemisphere, ‘Hass' avocado trees bloom in the early spring. Following fruit set in the spring, avocado fruit growth proceeds, with 8–12 months generally required before the fruit can be harvested [4]. In parallel, the shoots of avocado trees undergo at least two successive cycles of growth each year, referred to as the spring and summer flushes [4]–[6]. Although the vegetative shoots of each of these flushes have the potential to produce inflorescences, axillary buds of the spring flush typically primarily produce leaves or remain dormant, while the upper buds of the summer flush produce inflorescences. Specifically, the transition to flowering in ‘Hass' avocado is made possible only following exposure to low temperature conditions, namely a 3–4 month-long regimen of day/night temperatures of 10–15/15–18°C [5]. Consistent with this notion, changes at the macro-level that occur in the upper buds of summer-growing shoots, indicative of inflorescence initiation, are clearly detected in the northern hemisphere from January [4]. At the same time, controversy remains regarding definition of the ‘Hass' flowering induction period. While early studies suggesting that floral induction of ‘Hass' avocado grown in California occurs between late autumn and early winter [5], a later anatomical examination of ‘Hass' growing in the same region suggested that a transition from vegetative to the reproductive condition occurs in early summer [7]. Finally, it should be noted that although different physiological studies of ‘Hass' clearly indicate that the high yield obtained in an *on* year causes an attenuation of shoot elongation and decreases inflorescences number [7]–[9], the mechanism(s) by which avocado fruit load affects shoot growth and flowering are still poorly understood.

The transition from vegetative into reductive structure is one of the key developmental processes of flowering plants. Molecular evidence suggests that although flowers of different species are extremely diverse, a universal set of genes control floral induction and flower initiation in response to external signals (such as temperature change), and/or endogenous factors (such as changes in sugar or hormone availability). For instance, proteins similar in structure to the *Arabidopsis* FLOWERING LOCUS T (FT) that functions as a signal that moves from the leaves to the shoot meristem to mediate the floral transition have been identified in various plant species [10]. FT belongs to a small group of proteins that shows structural similarities to mammalian phosphatidylethanolamine-binding protein (PEBP) [10], [11]. In *Arabidopsis*, this gene family comprises six members (*FT, TWIN SISTER OF FT* (*TSF*) [12], *MOTHER OF FT* (*MFT*) [*13*] *, ARABIDOPSIS THALIANA CENTRORADIALIS HOMOLOGUES* (*ATC*) [14], *TERMINAL FLOWER 1(TFL1*) and *BROTHER OF FT* (*BFT*) [15]) encoding very similar proteins. While *AtTSF* and *AtMFT* function redundantly with *AtFT* to promote flowering [12], [13], *AtTFL1, AtATC* and *AtBFT* play an antagonistic role to that of *FT,* acting as floral inhibitors [14], [15]. Interestingly, the opposite functions of FT-like and TFL1-like proteins map only to two single amino acids encoding in the second exon and to a small external loop domain in the 4th exon of these genes [15]–[17].

In the shoot meristem, FT associates with the b-ZIP transcription factor FD to mediate floral transition by activating expression of MADS box genes, including *APETALA1 (AP1)*, *FRUITFULL (FUL)* and *SUPPRESSOR OF OVEREXPRESSION OF CONSTANS1* (SOC1) [18]. In turn, these MADS box transcription factors positively regulate floral meristem identity genes such as *Leafy (LFY)*, encoding a unique plant transcription factor necessary for flower formation [19]. While FT-mediated up-regulation of *AP1* results in irreversible transition into a reproductive meristem, a strong *LFY* expression that occurs rather late in the meristem, is suggested to reflect both the quantity and the quality of the different flowering signals perceived by the plant [19], [20].

Homologs of *Arabidopsis* flowering-related genes have been identified in various fruit tree species and in some cases, functional evidence showing that these proteins affect flowering has been presented (for review, see [3]). For example, very early flowering was shown to be caused by over-expression of FT-encoding genes in citrus [21] and apple trees [22]. Likewise an early flowering phenotype can be achieved by reduced expression of a TFL1-like gene, as recently shown in pear [23]. Interestingly moreover, recent studies of citrus [24]–[26] and mango trees [27] demonstrated that fruit load modulates the expression of distinct flowering-related genes in both leaves and bud tissues. In avocado however, although several cDNA sequences exhibiting bona fide flowering-related genes characteristics were identified by the Floral Genome Project (http://www.floralgenome.org/) and the Ancient Ancestral Genome Project (http://ancangio.uga.edu/) [28], description of their spatial and seasonal expression profiles during *off* and *on* years remains lacking. Indeed, it has yet to be established whether a factor such as a *FT-like* gene is also involved in the regulation of avocado inflorescence/flower induction, and/or whether fruit load modulates the expression of this putative gene. With this in mind, we here aimed to determine when and how high fruit load interferes with the ‘Hass' flowering process. To do so, we first performed fruit thinning experiments which helped define a physiological window of time between late autumn and early winter during which the negative effect of fruit load upon return to flowering was irreversible. Next, we isolated an avocado cDNA encoding a FT-like protein and confirmed its flowering regulatory function by its ectopical expression in *Arabidopsis*. Finally, using molecular markers and histological approaches, we showed that under local growth conditions, ‘Hass' inflorescence initiation take place during early winter, following a strong accumulation of an FT-encoding gene, which only occurs in the leaves of *off* trees. Possible mechanisms by which fruit load might repress *PaFT* expression, thus affecting return flowering and AB trait, are discussed.

## Materials and Methods

We thank Kibbutz Eyal for providing access to their ‘Hass' avocado orchard.

### Plant material

Mature commercially-bearing 7-year-old ‘Hass' avocado trees grafted on ‘Degania 117' rootstocks were used for all experiments. The experiment was conducted in the orchards of Kibbutz Eyal, located in the central district area of Israel (32°12N, 34°58 E), ‘Hass' trees grown in this orchard exhibited marked alternate-bearing behavior.

### De-fruiting treatments

Fruit load intensity of trees within the orchard was determined in early June, 2011. Twenty-four uniform heavily producing trees were selected for the experiment. The trees were randomly assigned to different de-fruiting treatments in groups of four trees (replicates) per treatment (different dates of fruit removal). Accordingly, manual complete de-fruiting treatments were performed at the onset of July, August, September, October or November, 2011. In the sixth (control *on*) treatment, fruits were collected during the commercial harvest season (February, 2012).

Shoot growth parameters were collected at various time intervals from August, 2011 until April, 2012 (the end of the flowering period). Ten new summer growing shoots were tagged on each tree at the beginning of August. At each interval, shoot lengths were measured from the tip of the shoot to the beginning of the summer flush, a point clearly defined by a cluster of closely spaced buds [4].

A fate map of buds along the shoot was produced based on data accumulated at the end of the flowering period. For this purpose, the apical buds and adjacent 7–8 axillary buds along the summer-growing shoots were assigned a status as 1, representing a bud that gave rise to vegetative structures (leaves), 2, representing a bud that gave rise to an inflorescence, and 3, representing a bud that remained dormant. The intensity of return flowering was evaluated the following spring, beginning March, 2012, using a blind test in which two surveyors independently scores each tree from 0 (no flowering) to 3 (high flowering intensity).

The total yield of fruit that developed from the flowers of March, 2012 was determined in treated (de-fruited) and untreated (*on*) trees, in February, 2013 by weighing all of the fruits harvested from individual trees.

### Plant tissue sampling

Six additional uniform heavily producing trees were selected in early June, 2011 for plant tissue sampling. The trees were randomly divided into two treatment groups, control and early fruit removal, with three trees (biological repeats) being subject to each treatment. The control trees were not thinned and fruit was harvested during the commercial harvest season. The early fruit removal treatment was performed at the beginning of July, 2011 by removing all fruitlets on the trees, thus mimicking the *off* condition. Subsequently tissue sampling was carried out early in the morning, at various intervals during the 2011/2012 season. At each interval, three vegetative shoots were pruned from the southern portion of the trees, collected and transported to the laboratory in buckets of water, where plant tissues were dissected. To maintain a uniform sampling pattern, the dissection of leaves, leaf petioles and shoot sections was performed starting from the fourth leaf relative to a reference point, set as the base of the summer vegetative flush. Finally, apical buds and the adjacent 3–5 axillary buds were also dissected and collected. Upon dissection, tissue samples were immediately frozen in liquid nitrogen and maintained at −80°C until further analysis.

### RNA isolation and cDNA synthesis

Total RNA from leaves, leaf petioles and shoot tissues was extracted using a phenol-SDS method [29]. Total RNA from buds samples was extracted using a Plant/Fungi Total RNA Isolation Kit (NorgenBiotekcrop, Thorold, Canada), following the manufacturer's instructions. RNA was quantified using a NanoDrop spectrophotometer, while RNA quality was verified by gel electrophoresis. Following confirmation of RNA integrity, 4 µg of total RNA, pre-treated with 1 unit of DNase, served as template in the synthesis of first strand cDNA using an oligo dT primer and M-MuLV reverse transcriptase (RT) kit (Fermentas). cDNA synthesis was conducted at 42°C for 60 min. RT was inactivated by boiling for 10 min. Real time-PCR (RT-PCR) reactions were next performed using RT reaction products, as described below.

### Isolation and cloning of *PaFT* cDNA by RACE-PCR

A search for avocado sequences encoding a protein similar to the product of the *FLOWERING LOCUS T* gene was performed using a nucleotide BLAST program against the Sequence Read Archive (SRA) database (http://www.ncbi.nih.gov/sra) and conserved regions of *FT* RNA as query. This search led to the identification of several overlapping sequences whose translated versions revealed best matches to the C-terminal part of *AtFT*. The longest read, which apparently also comprised the 3′ untranslated region (UTR) of the gene (SRR039683.132), was selected and its sequence was first verified by RT-PCR, using cDNA synthesized from *off* leaves (a mix of samples collected at different intervals) as template, together with PaFT1- and PaFT2-specific primers (see Table 1). The sequence of the obtained PCR product was further used to isolate full-length *PaFT* cDNA using a 5′/3′ RACE system (CapFishing kit, Seegene, Seoul, Korea). Accordingly, the 5′ end of *PaFT* was amplified with PaFT-3(AS1) and PaFT-4(AS2-nested) primers. Finally, the full-length cDNA of the *PaFT* gene was amplified using specific end-to-end primers, designed within the 5′ and 3′ UTRs of the gene. The primers used were PaFT-6 (S) and PaFT-5(AS) (Table 1). The obtained PCR product was ligated into the pGEM-T Easy vector (Promega), sequenced (Hy-labs Laboratories, Rehovot, Israel) and further used as template to create a *PaFT* standard curve for RT-PCR analysis, and to generate a construct for *Arabidopsis* transformation purposes (see below).

**Table 1:** Primers used in this study.

Amplified	Primer	Sequence (5′ to 3′)	Direction
Gene			
***PaFT***	PaFT-1	5' TACTTGCACTGGTTGGTGACAGNT 3'	S
	PaFT-2	5' TTTGGGCAGGAGATCGTCTGCTAT 3'	AS
	PaFT-3	5' CAATGCGAAGACCAGCCGATGAAT 3'	AS
	PaFT-4	5' ATAGCAGACGATCTCCTGCCCAAA 3'	AS
	PaFT-5	5' AAGAGGGAGAAAGAGTAGCAGTCC 3'	AS
	PaFT-6	5' CCAGCACTCGTTGTTGAGAGTGT 3'	S
	PaFT-7	5' ACTTCAACACCAGGGACTTTGCAG 3'	S
	PaFT-8	5' TAATAAGTTCTCCGGCTGTCGTCG 3'	AS
	PaFT-*EcoRI*	5' AAGTGAATTCCCAGCACTCGTTGTTGAGAGTGT 3'	S
	PaFT-*Bam*HI	5' TTCTGGATCCAAGAGGGAGAAAGAGTAGCAGTC 3'	AS
***PaAP1***	PaAP1-1	5' TCTGAGGGAAACTGGTGCCAAGAA3'	S
	PaAP1-2	5' GCTTCAACAGCTGGAACAACAGCT3'	S
	PaAP1-3	5'CGGATTATTTTTCTTCTTCTTCTCCT3'	S
	PaAP1-4	5' GCAAGATGTGTGCCAAAGAC 3'	AS
	PaAP1-5	5' GTAGCAGCAGAAGAGGAAGTAG3'	S
	PaAP1-6	5' GAGAGAGAGCGAGACCATCTA3'	AS
***PaLFY***	PaLFY-1	5' GCAGCGTGAACATCCCTTCATTGT 3'	S
	PaLFY-2	5' TGGATCAAGAACTCCCTGCACTGT 3'	AS
***PaACT***	PaACT-1	5' TGAAATACCCCATTGAGCATGG 3'	S
	PaACT-1	5' GAATCCAGTACAATACCTGTGGTACG 3'	AS

### Cloning of *PaAP1* and *PaLFY* cDNAs

An annotated EST sequence (accession number (ac) DQ398015), whose translated version revealed best match with a partial coding sequence encoding a putative avocado AP1 protein, was used to isolate full-length *PaAP1* cDNA. This sequence, lacking a C-terminal region and a 3′ end, was employed to design specific primers. Accordingly, PaAP1 3′UTR was amplified using a 5′/3′ RACE system, specific PaAP1-1(S1) and PaAP1-2 (S2-nested) primers and cDNA synthesized from *off* bud tissues (a mix of samples collected at different intervals). Next, full-length *PaAP1* cDNA was amplified using PaAP1-3 (S) and PaAP1-4 (AS) end-to-end primers.

Additionally, a BLAST search against the *Persea americana* expressed sequence tag (EST) database (http://www.ncbi.nih.gov), led to the identification of a partial coding sequence encoding a putative avocado LFY protein (ac: FD502004.1). This EST annotation was first verified by RT-PCR, using the specific PaLFY-1 (sense) and PaLFY-2 (anti-sense) primers (Table 1), and cDNA synthesized from *off* bud tissues. Both full length *PaAP1* cDNA and the *PaLFY* PCR product were ligated into the pGEM-T Easy vector, sequenced and used as template to create the corresponding standard curves for RT-PCR analysis (see below).

### Binary vector construction and *Arabidopsis* transformation

For constitutive expression of *PaFT* in *Arabidopsis*, the plasmid pART7-based pART27 vector was used [30]. The protein*-*coding region of *PaFT* was first amplified from the pGEM-T-*PaFT*-derived plasmid using PaFT-*Eco*RI and PaFT-*Bam*HI primers (Table 1). The purified PCR fragment was then digested with *Eco*RI and *Bam*HI and cloned into the *Eco*RI-*Bam*HI site of the pART7 vector between the *CaMV35S* promoter and the *ocs* 3′ transcription terminator. Next, the expression cassette including *CaMV35S*, *PaFT* and *ocs* 3′ was *Not*I-excised from the pART7 construct and inserted into the binary plant transformation vector pART27. The resulting plasmid, named pART27 35S::*PaFT,* was further used for stable transformation of wild type (Col-0) *Arabidopsis* plants using the *Agrobacterium tumefaciens*-mediated floral dip method [31]. The transformed seed were selected on medium containing half-strength Murashige and Skoog salts and kanamycin (50 µg/mL). The second generation of four randomly selected transgenic lines (13–20 plants per line) was used for phenotypic assessment of flowering times, along with wild type plants serving as controls. Plants were placed in growth rooms under a long day (25°C, 16/8 light/dark) or short day (25°C, 8/16 light/dark) regime. Flowering times were measured by counting the number of rosette leaves and the number of days from sowing until the first flower bud was seen.

### RT-PCR and primer design

The accumulation of *PaFT, PaAP1* and *PaLFY* genes in the sampled tissues was evaluated by RT-PCR using Absolute QPCR SYBR Green ROX Mix (ABgene, Epson, UK). Reactions were carried out using 3 µl of cDNA products (1∶10 dilution), 6 µl of SYBR Green PCR Master mix and 200 nM primers from the relevant primer pair, in a final volume of 12 µl. Analysis was performed in a Rotor GENE 6000 instrument (Corbett Life Science, Sydney, Australia). A dilution series of pGEM-T Easy plasmids, containing full length *PaFT* or *PaAP1* cDNAs or a partial *PaLFY* cDNA-amplified fragment, were created and standard curves for each gene were established using pairs of relevant primers (see below). cDNA samples were analyzed in triplicate, with each reaction being subjected to melting point analysis to confirm the presence of single amplified products. Transcript levels in each sample were estimated using a standard curve for each gene and normalized against the *PaACT* transcript level. Relative expression (RE) levels were calculated by dividing each individual gene copy number by the *PaACT* copy number. For each gene, RE levels were obtained after setting the lowest copy number value as 1. The primers used were: PaFT-7 (sense) and PaFT-8 (anti-sense), directed against the C-terminal part of the *PaFT* and its 3′UTR, for *PaFT*; PaAP1-5 (sense) and PaFT-8 (anti-sense), directed against the C-terminal part of the *PaAP1* and its 3′UTR region, for *PaAP1*; PaLFY-1 (sense) and PaLFY-2 (anti-sense), selected based on EST annotation FD502004.1, for *PaLFY* and PaACT-1 (sense) and PaACT-2 (anti-sense), for *PaACT* (GU272027) (Table 1).

### Anatomical studies

Apical buds, collected at various intervals from fully loaded (*on*) and completely de-fruited (*off*) trees, were fixed in FAA solution (10% (v/v) formaldehyde, 5% (v/v) acetic acid, 50% (v/v) ethanol, 35% (v/v) H_2_O), and dehydrated in a graded ethanol series. Samples were embedded in wax and cut using a microtome (Leica RM2245) into 12 µm-thick sections. The sections were stained with safranin and fast green, and examined under a light microscope (Olympus BX50).

### Statistical analysis

ANOVA testing of obtained data was conducted using JMP software, version 7 (SAS Institute, Cary, NC).

## Results

### Exploring the negative effects of crop load on shoot elongation and return to flowering

As mentioned above, previous studies on ‘Hass' avocado showed that heavy fruit load represses both shoot growth and return to flowering [7]–[9]. To explore the negative effect of fruit load on these two parameters in our growth conditions, and to help define the latest date at which fruit can still be removed and allow return to flowering, de-fruiting experiments were first performed. Complete fruit removal treatments were performed at the early and middle stages of fruit development and shoot elongation parameters and flowering intensity levels were next recorded.

**Figure 1:**
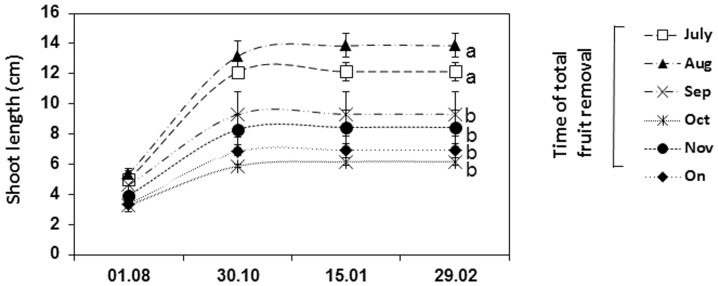
Effect of fruit load on shoot elongation. Complete fruit removal treatments, conducted from July, 2011 through November, 2011, were performed at the onset of each month. Shoot length records of treated (de-fruited) and untreated (*on*) trees were determined at various intervals. Values represent means±SE of 40 mesurments (10 shoots × 4 trees per treatment) and different letters indicate significant differences. Measuring dates are presented as dd/m.

The obtained shoot records indicated that “summer” vegetative growth occurred mainly from August to October (Fig. 1). Additionally, and in line with previous reports [7], [8], it was observed that heavy fruit load suppressed shoot growth. Accordingly, the final shoot length was significantly lower in control *on* trees, as compared to trees which were de-fruited in July and August, yet, no significant difference in shoot length were detected between *on* trees and trees which were de-fruited from September and on.

**Figure 2:**
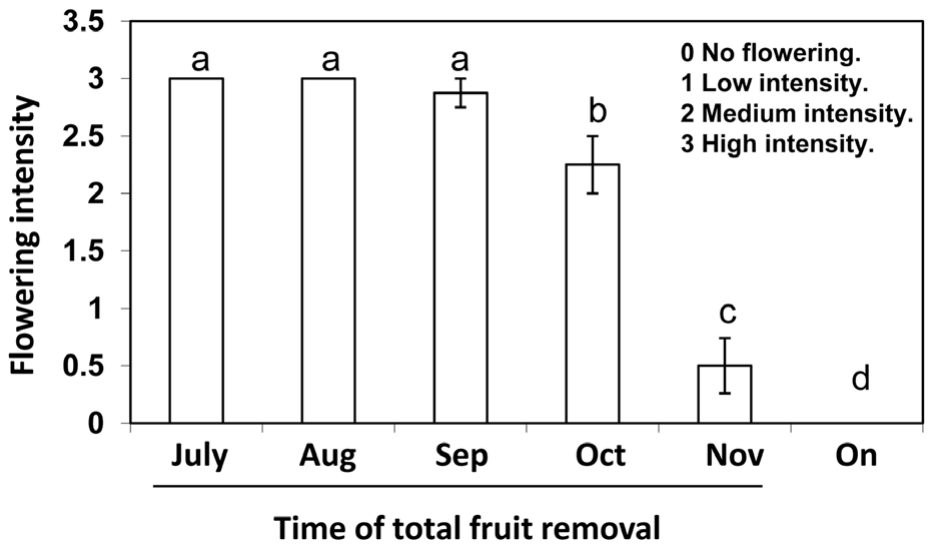
Effect of fruit load on return to flowering. Complete fruit removal treatments, starting in July, 2011, were performed as described in the legend to Fig. 1. Flowering-intensity levels of treated (de-fruited) and untreated (*on*) trees were ranked at the onset of the following spring (March, 2012). Values represent means±SE of four trees per treatment and different letters indicate significant differences.

Furthermore, the flowering records demonstrated that, as expected, control *on* trees whose fruits remained on the trees until the commercial harvest season displayed the most drastic reduction in flowering levels (Fig. 2). By contrast, treated de-fruited trees presented the highest degree of return to flowering, with the magnitude of return to flowering depending greatly upon the timing of fruit removal. When the fruit crop was removed before October, at different dates within the period spanning July to September, flowering occurred the following year, yielding a high blooming score. On the other hand, if fruit remained on the tree until October or later, fruit removal did not prevent the inhibitory effect of high fruit load on the next year’s flowering. Specifically, fruit removal at the onset of November resulted in a low flowering score that was, nevertheless, significantly higher than the null score of control *on* trees. Taken together, the above results suggest that the inhibitory effect of fruit load on shoot growth correlates with fruit growth, whereas the actual negative effect of fruit load on return to flowering only begins in October.

### Mapping the fate of meristems based on their position along the shoot

**Figure 3:**
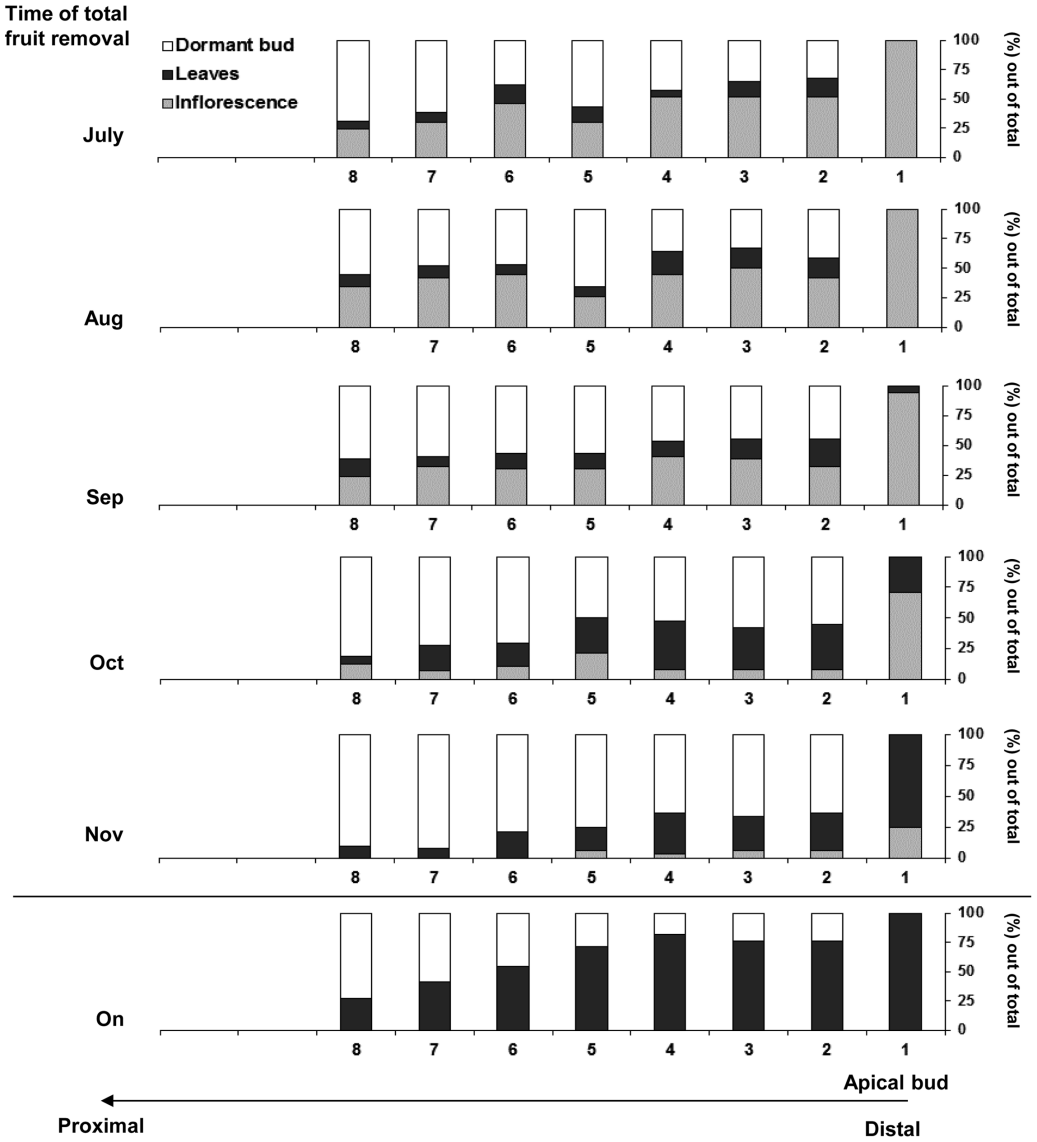
Effect of fruit load on inflorescence development. Complete fruit removal treatments, starting in July, 2011, were performed as described in the legend to Fig. 1. The reproductive fates of individual buds on treated (de-fruited) and untreated (*on*) trees borne along summer-growing shoots were mapped at the end of the flowering period (May, 2012). Values are expressed as percentage of dormant buds, leaves or inflorescences out of 40 records (of 10 shoots per tree) collected per treatment.

When examining the fate of buds in relation to heavy fruit load, it would be incorrect to generalize since the fate of an individual bud could depend on its relative position on the shoot. Earlier studies indicated that the vast majority of ‘Hass' inflorescences are produced on summer flush vegetative shoots [6], [7]. Specifically, it was observed that on summer-growing shoots, floral competency appears to decrease in a basipetal (i.e. top to bottom) direction. As such, apical and the most distal axillary buds usually give rise to inflorescences, whereas older buds tend to develop less inflorescences and/or remain dormant (field observations and H.M. Smith, personal communication). With these observations in mind, we carefully mapped the reproductive fate of individual buds borne along tagged summer growing shoots so as to further explore the negative effects of crop load on return to flowering. The results obtained demonstrated that transition from vegetative to reproductive structure was completely repressed in apical buds of *on* shoots, with young leaves appearing instead (Fig. 3). By contrast, 100% of the apical buds of shoots which were de-fruited in July produced inflorescences. Additionally, in all fruit removal treatments, the fate of an apical bud was well correlated with the overall flowering rate (Fig. 3). Specifically, fruit removal in early November allowed only 25% of apical meristems to flower.

Next, when studying the fate of axillary buds along the shoots, it was observed that the rate of flowering was lower, independent of treatment, and that the percentage of dormant buds increased with distance from the apical meristem, reflecting a classical effect of apical dominance. Nodes as far as the seventh node from the apical bud could become floral if fruit removal treatments were performed until the beginning of September. Fruit removal at the beginning of October allowed a certain degree of inflorescence formation until the eighth node, while fruit removal at the beginning of November limited the number of nodes capable of producing inflorescences to the first five nodes. From these results, it appears that the overall strength of the floral signal is affected by two parameters, distance from the apex and date of fruit removal.

### Fruit yield as compared to flowering intensity

**Figure 4:**
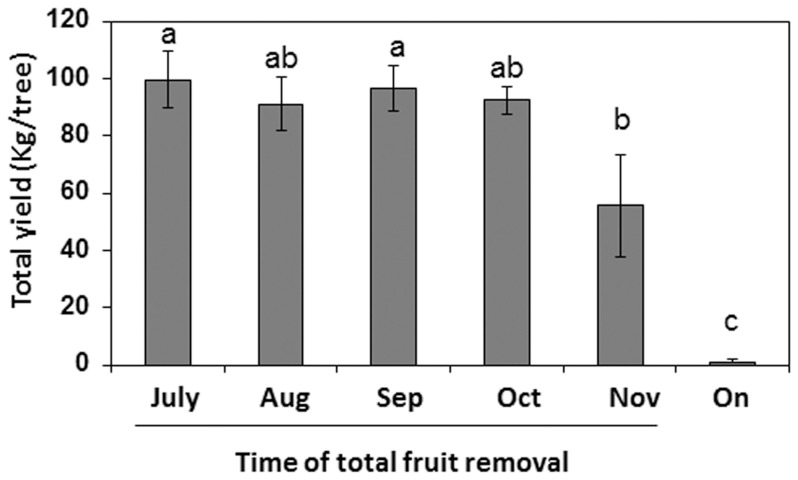
Effect of fruit load on fruit yield in the following year. Complete fruit removal treatments were performed as described in the legend to Fig. 1. Total yield per tree was determined in the following commercial harvest season (February, 2013). Values represent means±SE of four trees per treatment and different letters indicate significant differences.

The above data support the concept that the phenomenon of AB in ‘Hass' avocado grown in our conditions is associated with yearly changes in the degree of flowering. However, while intense flowering is certainly a prerequisite for fruit setting, it cannot guarantee fruit yield in the next season. Thus, to explore the relationship between flowering and next season yield, we determined the total yields of individual control (*on*) and treated (de-fruited) trees. The total yield of individual tree was determined during the harvest season (February, 2013) that follows the spring bloom (March-April, 2012). Fig. 4. shows that in individual control (*on*) trees, next year yield was negligible, as expected from the negligible amount of inflorescences. By contrast, the following year's yield from trees that were subjected to fruit thinning treatments between July and September was high and matched the level of flowering intensity. Interestingly, the reduction in flower intensity due to fruit removal in October and November had a less pronounced effect on final fruit yield. It thus appears that the effect of fruit load on flowering was more extreme than was its effect on the next year’s yield. This is likely due to the fact that with fewer inflorescences, the fruit set and fruit retention rate increase. Also, with less fruit, the size of each fruit may increase, leading to a lower overall negative effect on total yield. Indeed, in trees from which fruits were removed at the onset of November, large size fruits (>220g) accounted for a higher fraction of the total yield, as compared to other treated trees (Fig. S1). Assuming that a reduction in the level of flowering intensity brought a reduction in the number of fruit per tree, the increased size of such fruit could contribute to the elevation of the averaged total yield.

### Isolation of a *FLOWERING LOCUS T* (*FT*) homolog from ‘Hass' avocado

**Figure 5:**
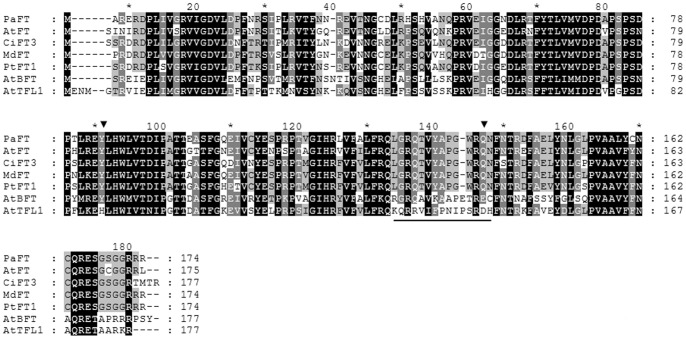
Amino acid sequence comparison and structural features of avocado PaFT. Comparison of the deduced amino acid sequence of PaFT (ac: AIG92770) with four representative FT proteins, including: *Arabidopsis* AtFT (ac: BAA77838.1), citrus (*Citrus unshiu*) CiFT3 (ac: BAA77836.1), apple (*Malus x domestica*) MdFT (ac: ACL98164.1) and populus (*Populus tremula*) PtFT1(ac: ABD52003.1), together with BFT and TFL1 proteins from *Arabidopsis*, AtBFT (ac: Q9FIT4.1) and AtTFL1 (ac: AED90661.1). Residues on black, dark gray and light gray backgrounds indicate 100%, 75%, and 50% amino acid similarity, respectively. Dashed lines indicate gaps introduced to achieve maximum alignment. Key FT residues are marked with triangles. PaFT Tyr-84 and Gln-139 correspond to AtFT Tyr-85 and Gln-140. The conserved amino acid sequence LGRQTVYAPGWRQN, which distinguish FT from TFL and/or BFT, is underlined.

Recent studies have suggested that proteins similar in structure to *Arabidopsis* FT might also act in perennial trees to promote floral induction (for reviews, see [3], [30]). Here, to test whether a similar *FT* gene is also found in avocado, a combined RT-PCR and RACE strategy was adopted. The obtained avocado *FT* clone contains an ORF of 855 bp, with a 3′ UTR of 127 bp and a 5′ UTR of 203 bp, which encodes a 174 amino acid residue protein. As shown in Fig. 5., CLUSTALW-based alignment of the putative translated FT protein from avocado reveals that the predicted protein shares 96–99% identity with FT protein from various plant species. Moreover**,** the predicted PaFT protein also contains major characteristics associated with FT activity, including two conserved amino acid that have been reported as key for promoting flowering, namely Tyr-84 and Gln-139, -corresponding to *Arabidopsis* FT Tyr-85 and Gln-140, as well as the conserved amino acid sequence LGRQTVYAPGWRQN, which distinguishes FT from TFL and/or BFT **-** proteins [15]–[17]. The sequence similarity of the predicted avocado FT protein to various FT-like proteins supports the identification of the isolated avocado clone as a gene encoding a FT protein. The identity of PaFT was also confirmed by phylogenetic analysis (see Fig. S2). According to phylogenetic analysis, the PaFT protein was more closely related to other FT and TSF proteins than to BFT or TFL proteins. Based on these observations, the isolated cDNA was termed *PaFT* and annotated as GenBank accession number KM023154.

### Examining the effect of ectopic expression of *PaFT* in transformed *Arabidopsis* plants

**Figure 6:**
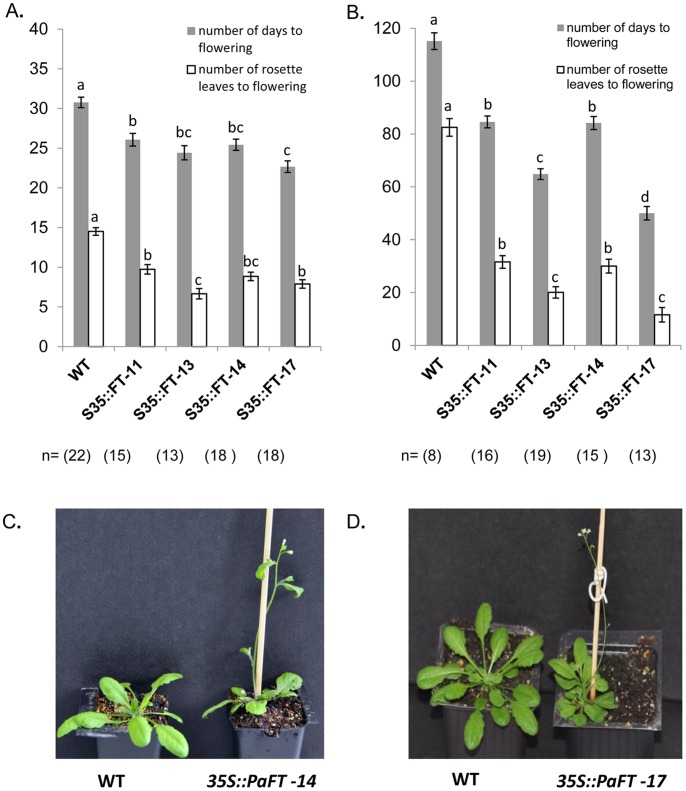
Ectopic expression of PaFT in *Arabidopsis*. Number of days to flowering and the number of rosette leaves at flowering of wild type plants and four randomly selected lines that constitutively expressed *PaFT* under LD (**A**) or SD (**B**) conditions are shown. Values represent means±SE of n = 8-22 plants per treatment and different letters indicate significant differences. Two randomly selected transforemed lines that constitutively expressed *PaFT* exhibiting early flowering phenotype relative to wild type plants under LD (**C**) and SD (**D**) conditions are shown. Photographs were taken after 22 (**C**) and 48 days (**D**).

As a first attempt in investigating the function of PaFT in flowering, its cDNA, driven by the cauliflower mosic virus (CaMV) 35S promoter, was ectopically expressed in wild type *Arabidopsis* Col-0 plants. After transforming with the pART27 35S::*PaFT* construct, 24 independent PCR-positive 35S::*PaFT* kanamycin-resistant plants were selected. All selected plants were phenotypically distinguishable from wild type plants and exhibited an early flowering phenotype under inductive long day (LD) conditions (not shown). Next, the transformed plants were self-pollinated and seeds from four randomly selected transgenic lines were sown directly on soil, cultivated under LD and short day (SD) conditions and used for a detailed phenotypic analysis, together with non-transformed control plants. Roughly, transgenic lines of the second generation grown in either day length conditions exhibiting an early flowering phenotype, segregated in a ratio of 3∶1. Furthermore, in the four randomly selected transgenic lines, *PaFT* promoted early floral transition under both LD and SD conditions (Fig. 6). As such, under inductive LD conditions, transgenic lines flowered within 18–25 days of seed sowing after producing eight rosette leaves, whereas wild type control plants flowered within 31 days of seed sowing after producing 15 rosette leaves. Moreover, under non-inductive SD conditions, transgenic lines flowered within 52–83 days of seed sowing after producing 12–31 rosette leaves (different transgenic lines), whereas control plants flowered within 115 days from sowing after producing 82 rosette leaves. Except for accelerated flowering, no obvious alterations of floral organs were observed in the 35S::*PaFT* transgenic *Arabidopsis* plants (Fig. 6C,D).

### Monitoring the expression of *PaFT* as a molecular marker of floral induction

Following corroboration of the potential florigenic role of *PaFT* in transgenic *Arabidopsis,* to gain further insight into the role played by *PaFT* in avocado floral determination, its temporal expression patterns were next studied in tissues collected from of *on* (fully loaded) and *off-like* (July de-fruited) trees, shown to go through flower transition. Notably, in model organisms, *FT* encoding genes are expressed in leaves and only the FT protein seems to move towards the meristem. Interestingly however, in other species such as citrus and apple, transcripts of FT-encoding genes, suggested to be involved in flowering induction process, were also detected in buds [26], [32]. Thus, in order to avoid missing an increase in *PaFT* expression, we followed its expression patterns in four different tissues: leaf lamina, leaf petiole, stem and bud.

**Figure 7:**
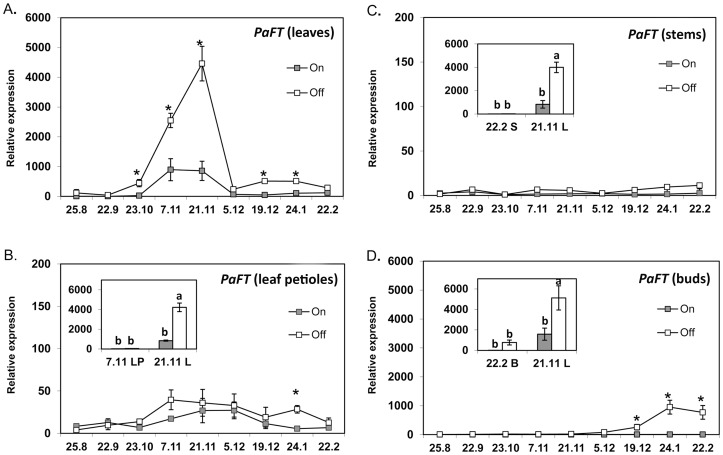
Seasonal expression profiles of *PaFT*. Seasonal expression profiles of *PaFT* in leaves (A), leaf petioles (B), stems (C) and buds (D) from fully loaded (*on*) and completely de-fruited (*off*) ‘Hass' trees are presented. *Off* trees correspond to trees from which fruit load was completely removed in July. Data are means±SE of three independent replicates (n = 3 trees). Stars denote a significant difference between transcript expression in *on* and *off* tissues at the same time point (*P*<0.05). Insets: *PaFT* expression in leaves (L) vs. leaf petioles (LP), (**B**) in leaves (L) vs. stems (S), (**C**) and in leaves (L) vs. buds (B) (**D**). Different letters indicate statistically significant differences (*P*<0.05). Sampling dates are presented as dd/m.

RT-PCR analysis revealed a strong increase in *PaFT* expression from the end of October through November in leaves of *off* trees, followed by a return to initial levels by the beginning of December (Fig. 7A). By comparison, *PaFT* mRNA followed similar kinetics in *on* leaves, except that from October through November, the *PaFT* transcript was significantly lower, relative to the level observed in *off* leaves. Interestingly, the initial increase in *PaFT* mRNA correlated with a decrease in temperature levels in both cases (Fig. S3).

A parallel examination of *PaFT* accumulation in leaf petioles and stem samples connecting leaves and buds revealed that *PaFT* mRNA levels remained stable and very low in both tissues almost throughout the sampling period (Fig. 7B,C). Moreover, a comparison of the expression levels of *PaFT* mRNA in leaves and leaf petioles and in leaves and stems, at those time points at which expression was maximal, revealed that in both cases, *PaFT* exhibited significantly higher expression levels in leaves (see Fig. 7B,C and insets).

Lastly, bud analyses revealed that while *PaFT* transcript levels in *on* buds remained low, they increased significantly in *off* buds from the beginning of December. Nevertheless, *PaFT* expression was substantially higher in leaves, as compared to buds (Fig. 7D and inset).

### Following the expression of LEAFY- and APETALA1-encoding genes in *on* and *off* tissues

Previous studies in fruit trees, including apple [32], citrus [33] and mango [27], showed that enhanced expression of *AP1 and LFY* genes, which follows an increase in *FT* transcript accumulation, might serve as useful molecular markers of flower initiation. As mentioned above, although cDNA sequences putatively encoding AP1 and LFY proteins from avocado have been deposited in distinct ESTs databases (http://www.floralgenome.org and http://ancangio.uga.edu/), these genes have thus far not been used as markers to define flower initiation processes. Interestingly however, results from a previous study on ‘Hass' avocado showed that the expression of a gene encoding an AP1 protein (termed *PaAP1*) is almost as high in leaves as in sepals/tepals and petals, thus suggesting that this gene does not encode a flower-specific organ identity gene [28].

**Figure 8:**
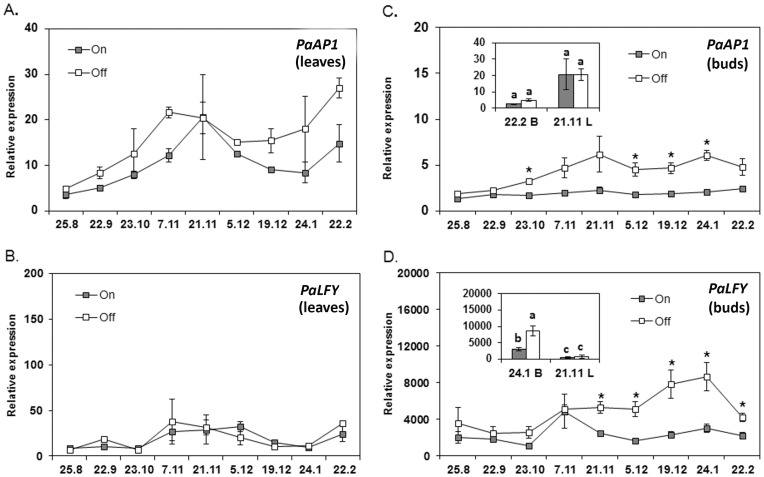
Seasonal expression profiles of *PaAP1* and *PaLFY*. Seasonal expression profiles of *PaAP1* and *PaLFY* in leaves (A–B) and buds (C–D) from fully loaded (*on*) and completely de-fruited (*off*) ‘Hass' trees are shown. *Off* trees correspond to trees from which fruit load was completely removed in July. Data are means±SE of three independent replicates (n = 3 trees). Stars denote a significant difference between the transcript expression in *on* and *off* tissues at the same time point (*P*<0.05). Insets: *PaAP1* and *PaLFY* expression in leaves (L) vs. buds (B) (**C** and **D**), respectively. Different letters indicate statistically significant differences (*P*<0.05). Sampling dates are presented as dd/m.

Here, to assess whether *PaAP1* and/or *PaLFY* expression patterns might help define a ‘Hass' inflorescence initiation process, and to correlate between *PaFT* expression and inflorescence commencement, we examined *PaAP1*and *PaLFY* expression in plant tissues (leaves and buds) that were used to monitor *PaFT* transcript levels. RT-PCR leaf analysis showed that throughout the examination period, both o*ff* and *on* leaves displayed a moderate up-regulation of *PaAP1* transcription, while *PaLFY* mRNA levels remained stable and low in both *off* and *on* leaves throughout the entire sampling period (Fig. 8A,B). Furthermore, bud analyses revealed that only *off* buds displayed substantial up-regulation of *PaAP1* and *PaLFY* transcription, which became significant at the end of October for *PaAP1* and at onset of December for *PaLFY* (see Fig. 8C,D). Finally, a comparison of the expression levels of flowering identity genes in leaves and buds revealed, that as expected, *PaAP1* accumulation, did not significantly differ between leaves and buds (Fig. 8C, inset). *PaLFY* accumulation, on the other hand, exhibited substantial higher expression levels in bud as opposed to leaf tissues (Fig. 8D, inset), suggesting *PaLFY* to be a good marker for avocado inflorescence initiation.

### Using histological approaches to time inflorescence initiation in avocado ‘Hass' trees

**Figure 9:**
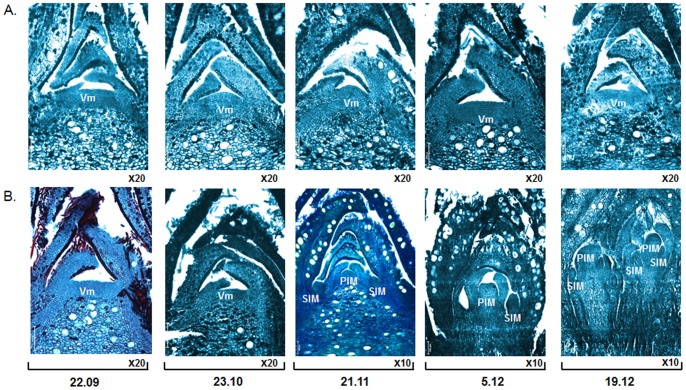
Successive microscopic views of apical buds. Successive microscopic views of apical buds were collected at different times from *on* (A) and *off* (B) trees. *Off* trees correspond to trees from which fruit load was completely removed in July. Abbreviations: VM, vegetative meristem; PIM, primary inflorescence meristem; SIM, secondary inflorescence meristem.

The above presented data associates *PaFT* with avocado flowering induction taking place in early winter, with the increase of *PaFT* occurring in leaves and the presence of fruit severely dampening this increase. As an additional feature, to help associate between *PaFT* up-regulation observed in *off* tissues and ‘Hass' inflorescence development, we carried a microscopic study of apical buds collected from *off* (fruit-lacking) trees. A parallel study was conducted using apical buds collected from *on* trees, as a control. Fig. 9 shows successive microscopic images of *off* and *on* apical buds collected from September to November. As predicted, and in agreement with the obtained flowering data, the microscopic analysis revealed that only *off* buds displayed secondary and tertiary axis inflorescence structures, which typically form inflorescences. Moreover, the presence of secondary inflorescence axis structures only became visible towards the end of November (Fig. 9A,B), when *PaFT* transcript levels in leaves peaked, concomitant with the initial increase in *PaLFY* mRNA levels.

## Discussion

### Fruit load and return to flowering

Alternate bearing has a major impact on fruit/nut tree crop productivity. The mechanism(s) controlling AB are poorly understood, making it difficult to develop approaches to mitigate this problem. In the classical and most common version of AB, the presence of fruit on a shoot causes a cessation of shoot growth and decreases the ability of the shoot to undergo floral transition the following spring [1], [2], [3]. Still, there are few cases in which AB occurs despite adequate return to flowering. In such cases, the dynamics of flower and fruitlet abscission are influenced by the production of a previous heavy *on* crop, affecting yield in the next season [1]. Results from a previous study addressing the nature of AB in ‘Hass' avocado provided convincing evidence showing that a preceding year's yield did not alter the next season’s flower and fruitlet abscission processes [34]. Furthermore, various field observations, as well as fruit removal studies, performed during the summer months in California indicated that in ‘Hass' avocado, a heavy *on* crop repressed return to flowering [4]. In agreement with these reports, results from our de-fruiting experiments confirmed, once again, the classical nature of the ‘Hass' AB trait and showed that heavy fruit load suppressed both shoot growth and return to flowering. Additionally, our next season yield assessments overall also confirmed the existence of a positive relationship between high flowering score and high fruit yield.

Note, that the inhibition of shoot growth by fruit load might reduce the contribution of those shoots to bloom by reducing axillary bud number. In the present study, we did not determine the number of axillary buds along examined shoots, thus we could not ascertain this possibility. On the other hand, our results showed that while a high fruit yield on trees until September did not prevent return to flowering, the presence of fruit on shoots from October and later greatly reduced transition to flowering. As such, a critical physiological window of time (from October to November) from which onward fruit load effectively represses flowering transition appears to exist. In this context, it should be mentioned that similar observations were obtained following de-fruiting experiments performed with citrus and olive trees [24], [35]. Based on these observations, it was hypothesized that in fleshy fruit trees there is a point of no return from which irreversible molecular or metabolic events, modulated by fruit load, effectively suppress transition to flowering [24]. Our data fit in principal with this notion and suggest that in ‘Hass' avocado, these irreversible changes probably occur in early winter.

### 'Hass' avocado PaFT may assume a florigen function

The function of a key protein such as FT, hypothesized to act as a phloem-mobile florigen signal that controls transition to flowering, appears to be conserved among various plant species [10]. To date, information regarding FT-encoding genes from avocado was missing. Here, we have isolated a full length avocado cDNA encoding a putative FT protein (named *PaFT*). In line with its predicted function, ectopic expression of *PaFT* in *Arabidopsis* induced precocious flowering phenotypes, thus reinforcing the concept that PaFT acts as a component of the avocado flowering pathway, acting to trigger flower/inflorescences development.

It should be noted that expression studies conducted in various plant species have revealed the existence of several FT paralogs that sometimes exhibit different spatial and temporal expression patterns, and/or functions. For instance, characterization of two apple FT-encoding genes (*MdFT1* and *MdFT2*) showed that their expression patterns clearly differ. As such, in studies that were conducted in the Northern hemisphere, *MdF1* expression was shown to increase in the apical meristems prior to the flowering initiation stage that occurs in early summer [32], [36], whereas *MdFT2* was reported to peak at a later stage (in September and April), in both flower and fruit tissues [36]. Based on these observations, it was suggested that while *MdFT1* could play a role promoting flowering, the increase in *MdFT2* might be related to the development of floral and or fruit organs. On the other hand, in poplar trees, where two very similar FT paralogs exist (*PtFT1* and *PtFT2*), it has been shown that sub-functionalization of the two FT-like genes occurs. As such, *PtFT1* is predominantly expressed in leaves during winter, and is likely to be responsible for floral induction, while *PtFT2* is expressed in leaves during the vegetative growth period in the summer, and is suggested to be involved in regulating vegetative growth cessation [37]. In the present study, we were able to isolate only a single FT-like gene. Whether other avocado FT paralogs exist, remains to be established.

The isolation of *PaFT* allowed us to further investigate its spatial and temporal expression profile both prior to and during avocado flower/inflorescence development. Such analysis revealed a strong peak in *PaFT* expression in early winter in *off* leaves, followed by a return to initial levels. Furthermore and concomitant with inflorescence development, *off* buds also displayed a significant up-regulation of floral identity transcripts which parallels and/or follows (in the case of *PaAP1* and *PaLFY*, respectively) the initial increase of *PaFT* in leaves. The finding that substantial accumulation of *PaFT* mRNA occurred in *off* leaves at the same time point that was deemed critical for regulation of transition to flowering (from October to November) supports the notion that elevated *PaFT* could induce return to flowering. The possible link between *PaFT* and avocado flower induction was further supported by microscopic analysis of *off* buds, revealing the presence of secondary inflorescence axis structures only towards the end of November, when *PaFT* transcript levels in leaves peaked. Taken together, our data suggest a molecular mechanism through which increased *PaFT* expression, peaking in leaves during November, provokes flowering induction in early winter. We note that although this conclusion does not comply with current understanding which predicts that in California, ‘Hass' flowering induction period occurs in the mid- to late summer [7], our data are most consistent with the notion that in our conditions, ‘Hass' avocado flowering induction period occurs from October through November.

Furthermore, at the transcriptional level it was noted that *PaFT* was predominantly expressed in leaf tissues, as compared to other examined tissues, including leaf petioles, stems and buds. Remarkably however, o*ff* buds also displayed an ameliorated significant up-regulation of *PaFT* mRNA, which was detected from December and on. As mentioned earlier, given that elevated expression of FT-encoding genes in bud tissues associated with the flowering induction period, had already been reported in other fruit tree species [24], [26], our findings depicting a significant increase in *PaFT* mRNA in *off* buds were not surprising. Yet, one can still ask what function might PaFT fulfill in avocado buds, following the flowering induction period? Evidence from molecular evolution studies suggests that the emergence of FT-like genes coincide with the evolution of flowering plants, thus the role of FT in flowering promotion seems to be conserved in all angiosperms [10]. Nevertheless, recent studies have now shown that in addition to their predominant role in control of flowering timing, FT proteins might assume a broader range of functions, such as controlling different developmental processes, including growth [38], fruit set [39], dormancy release [40] and even tuber formation [41]. In line with these reports, the possible involvement of *PaFT* in the control of additional flower developmental processes occurring in buds before anthesis is conceivable.

Finally, it also should be mentioned that although general dogma holds that the FT protein acts as the main component of the phloem-mobile signal mediating floral transition [42], [43], the possibility that a transportable *FT* mRNA also contributes to systemic florigen signaling is still debatable [44]. Here, in theory, our results showing an up-regulation of *PaFT* mRNA in reproductive buds preceding the peak of *PaFT* in leaves could be attributed either to *de novo* transcription of *PaFT* in buds and/or to *cis*-transportation of cellular *PaFT* mRNA from leaves into buds. Nevertheless, since expression analyses failed to detect elevated *PaFT* transcript accumulation in either leaf petioles or stem tissues connecting between leaves and buds, the possibility that *PaFT* mRNA accumulation in a bud results from transport of the mRNA product from leaves into buds can likely be ruled out.

### Repression of *PaFT* expression by fruit load might affect the ‘Hass' AB trait

The isolation of *PaFT* allowed us not only to investigate its expression profile in trees lacking fruit load but also in trees bearing heavy fruit load (under *on* conditions). In the former condition, it was observed that fruit load repressed both the increase of *PaFT* in leaves and the increase of *PaAP1* and *PaLFY* transcripts in buds. Assuming that PaFT adopts a florigenic function, these results might suggest that fruit load negatively affects return to flowering by repressing *PaFT* expression in leaves, affecting in turn *PaAP1* and *PaLFY* up-regulation in buds. In addition, considering previous studies reporting on the repressed expression of FT-encoding mRNAs in leaves and/or buds of trees bearing heavy fruit load [24]–[27], the finding that a similar phenomenon occurs in avocado suggests that this might represent a general mode of action by which fruit load affects AB. The question that remains open, however, is how fruit load modulates FT-encoding gene expression.

Several external environmental cues and internal endogenic factors have been implicated in the control of *FT* genes expression in perennial trees. For instance, accumulation of *FT-*encoding genes in citrus stem [33] and in poplar leaves [37] were shown to be induced by low temperatures. Similarly, in mango, in which floral induction occurs during late autumn, up-regulation of an FT-encoding gene was observed only in leaves of adults trees, following expose to cool temperature [27], thus suggesting that this might be a common mechanism used by some woody perennials to control floral induction. Indeed, in line with this concept and consistent with the notion that ‘Hass' avocado return to flowering requires expose to low temperature conditions [4], results from our study showed that a decrease in ambient temperatures correlated with the up-regulation of *PaFT* mRNA. Moreover, since the sharp increase in *PaFT* mRNA levels was only noted in *off* leaves, this observation suggests a model in which *PaFT* expression is induced by low temperature, whereas heavy fruit load prevents recognition of this low temperature-based signal. Alternatively, other models involving fruit load, indirectly or directly, imposing changes in leaf nutritional and/or hormonal status and affecting *PaFT* expression, regardless of the sensing of a low temperature flowering-induction signal, can be envisaged.

Current knowledge suggests that developing fruit provides a strong sink for carbohydrates produced in leaves (dominant source tissues). At the same time, recent studies of *Arabidopsis* have provided several lines of evidence assigning roles for sugars in regulating flowering time and inducing *AtFT* expression [45], [46]. Over the last decades, the possibility that sugars play a flowering regulatory function in fruit trees has been controversial [47], [48]. Our results, however might fit a scenario whereby during *off* years, the absence of ‘Hass' avocado fruits (dominant sink tissues) would increase leaf sugar levels, with this inducing *PaFT* expression.

Furthermore, fruits and seeds are also known to produce plant hormones which might play regulatory role, controlling return to flowering. For instance, export of gibberellin (GA) from fruit seeds into buds has long being suggested to inhibit flowering in many fruit tree crops, including avocado [49], [50]. Moreover, recent studies performed with mango and citrus trees have indicated that GA may act to inhibit flowering by repressing FT-encoding mRNA accumulation in both leaves and buds [27], [51], yet, proof supporting GA movement from seeds into leaves and/or buds have not been provided. Similarly, auxin, which is known to be actively transported out of the developing fruit via the polar auxin transport system, was also suggested to act as a possible fruit exportable hormone that represses flowering induction [52]. In light of the above assumptions, the probability that hormone export from developing ‘Hass' avocado fruit into leaves and/or bud tissues, and/or the interplay between hormone signaling pathways controls *PaFT*, seems reasonable. An in-depth exploration of processes that control avocado flowering process is now required. Indeed, a better understanding of mechanisms by which fruit load affects the regulation of flowering related gene expression, might lead to development of reliable methods designed to mitigate AB of this important food crop.

## Supporting Information

Figure S1 **Effect of fruit load on next year total yield and fruit size distribution.** Complete fruit removal treatments, starting in July, 2011, through November, 2011, were performed at the onset of each month. The total yield of fruits developing from fruit setting in March, 2012, was determined in February, 2013 by weighing all fruits harvested from treated (de-fruited) and untreated (*on*) trees. Fruits were grouped into three categories, small fruit (SF), corresponding to fruit weighing less than 150 g, normal fruit (NF), corresponding to fruit weighing 150–220 g and large fruit (NF), corresponding to fruit weighing more than 220 g. For each tree, individual fruit groups were weighed separately, and data was plotted on two graphs (**A** and **B**). In the upper panel, each column represents the mean±SE of four trees per treatment and different letters indicate significant differences. In the lower panel, fruit size distribution was calculated as percentage of the total.
(TIF)

Figure S2 **Phylogeny of FT, TSF, MFT, TFL and BFT proteins.** Conserved amino acid sequences of *Persea american* FT protein (boxed) and FT, TSF, MFT, TFL and BFT proteins from various plant species were compared using the CLUSTALW multiple alignment program. The phylogenetic tree was constructed using the Neighbor-Joining method (http://www.genome.jp). Database accession numbers are given in parenthesis.
(TIF)

Figure S3 **The increase in** ***PaFT*** **mRNA levels correlates with a decrease in temperature levels**. Minimal and maximal temperatures recorded from mid-August until the beginning of March at the Tel Mond stiation near kibbutz Eyal, where experimental ‘Hass' trees were grown (**A**). The arrow denotes the initial date when the minimal temperature dropped below 15°C. Seasonal expression profiles of *PaFT* in leaves from fully loaded (*on*) and completely de-fruited (*off*) ‘Hass' trees are shown. *Off* trees correspond to trees from which fruit load was completely removed in July (**B**). Data are means±SE of three independent replicates (n = 3 trees).
(TIF)
