# Supplementary figures and images for: Expression Profiling of FLOWERING LOCUS T-Like Gene in Alternate Bearing ‘Hass' Avocado Trees Suggests a Role for PaFT in Avocado Flower Induction
Source: PLoS One. 2014 Oct 17;9(10):e110613. doi: 10.1371/journal.pone.0110613 (PMC4201567; doi:10.1371/journal.pone.0110613)

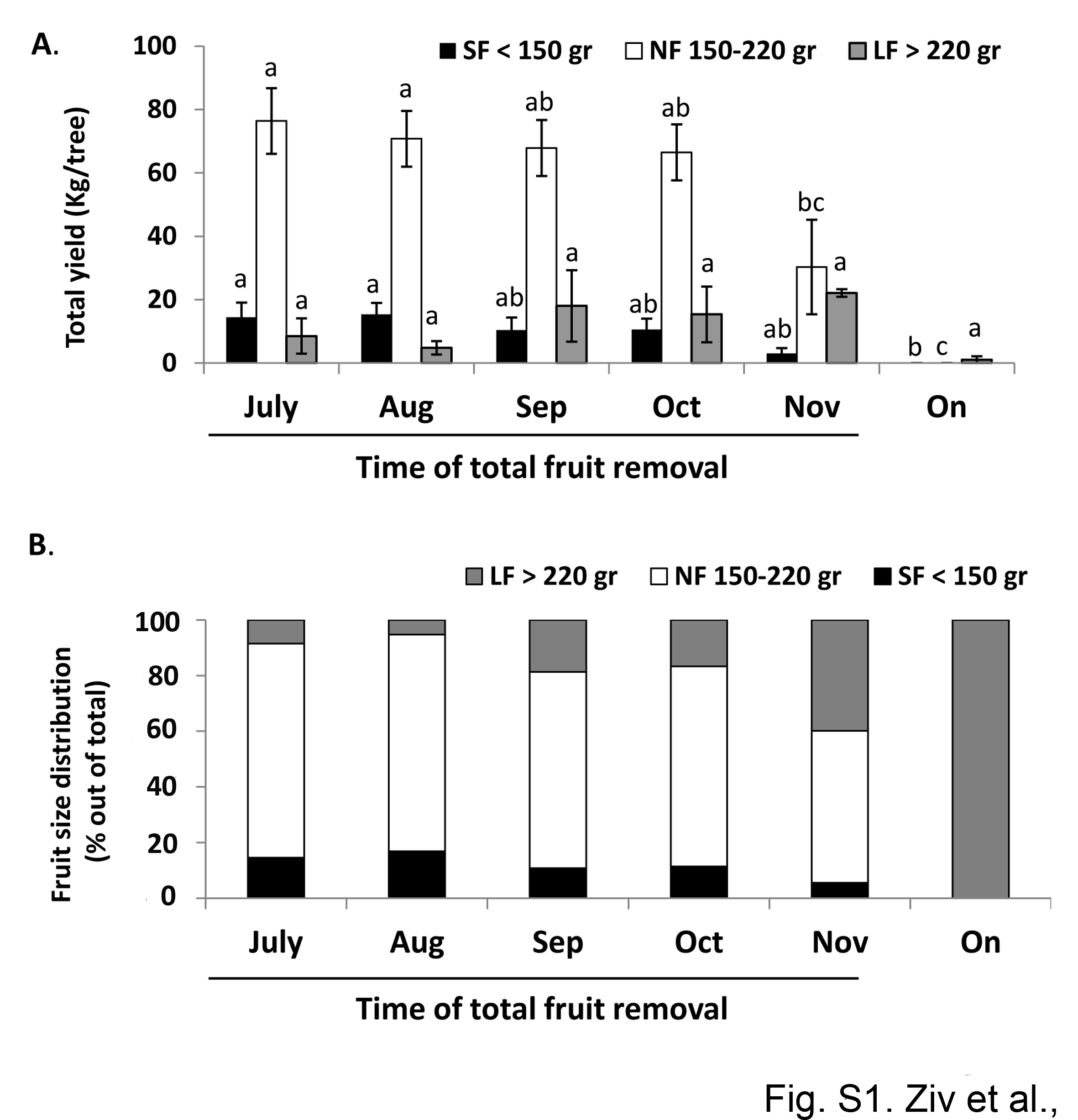

Supplement: Figure S1 — Effect of fruit load on next year total yield and fruit size distribution. Complete fruit removal treatments, starting in July, 2011, through November, 2011, were performed at the onset of each month. The total yield of fruits developing from fruit setting in March, 2012, was determined in February, 2013 by weighing all fruits harvested from treated (de-fruited) and untreated (on) trees. Fruits were grouped into three categories, small fruit (SF), corresponding to fruit weighing less than 150 g, normal fruit (NF), corresponding to fruit weighing 150–220 g and large fruit (NF), corresponding to fruit weighing more than 220 g. For each tree, individual fruit groups were weighed separately, and data was plotted on two graphs (A and B). In the upper panel, each column represents the mean±SE of four trees per treatment and different letters indicate significant differences. In the lower panel, fruit size distribution was calculated as percentage of the total. (TIF) [file pone.0110613.s001.tif]

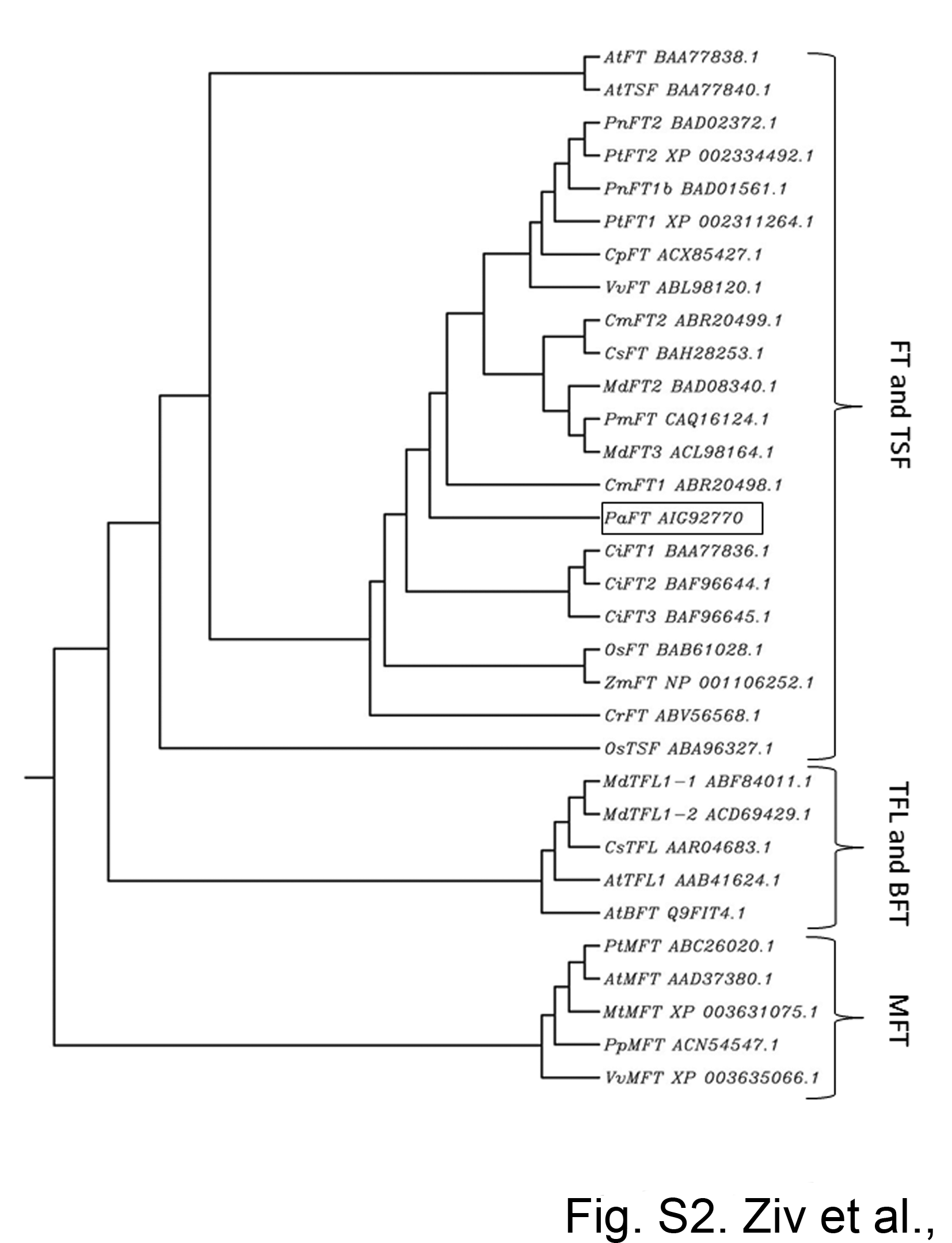

Supplement: Figure S2 — Phylogeny of FT, TSF, MFT, TFL and BFT proteins. Conserved amino acid sequences of Persea american FT protein (boxed) and FT, TSF, MFT, TFL and BFT proteins from various plant species were compared using the CLUSTALW multiple alignment program. The phylogenetic tree was constructed using the Neighbor-Joining method (http://www.genome.jp). Database accession numbers are given in parenthesis. (TIF) [file pone.0110613.s002.tif]

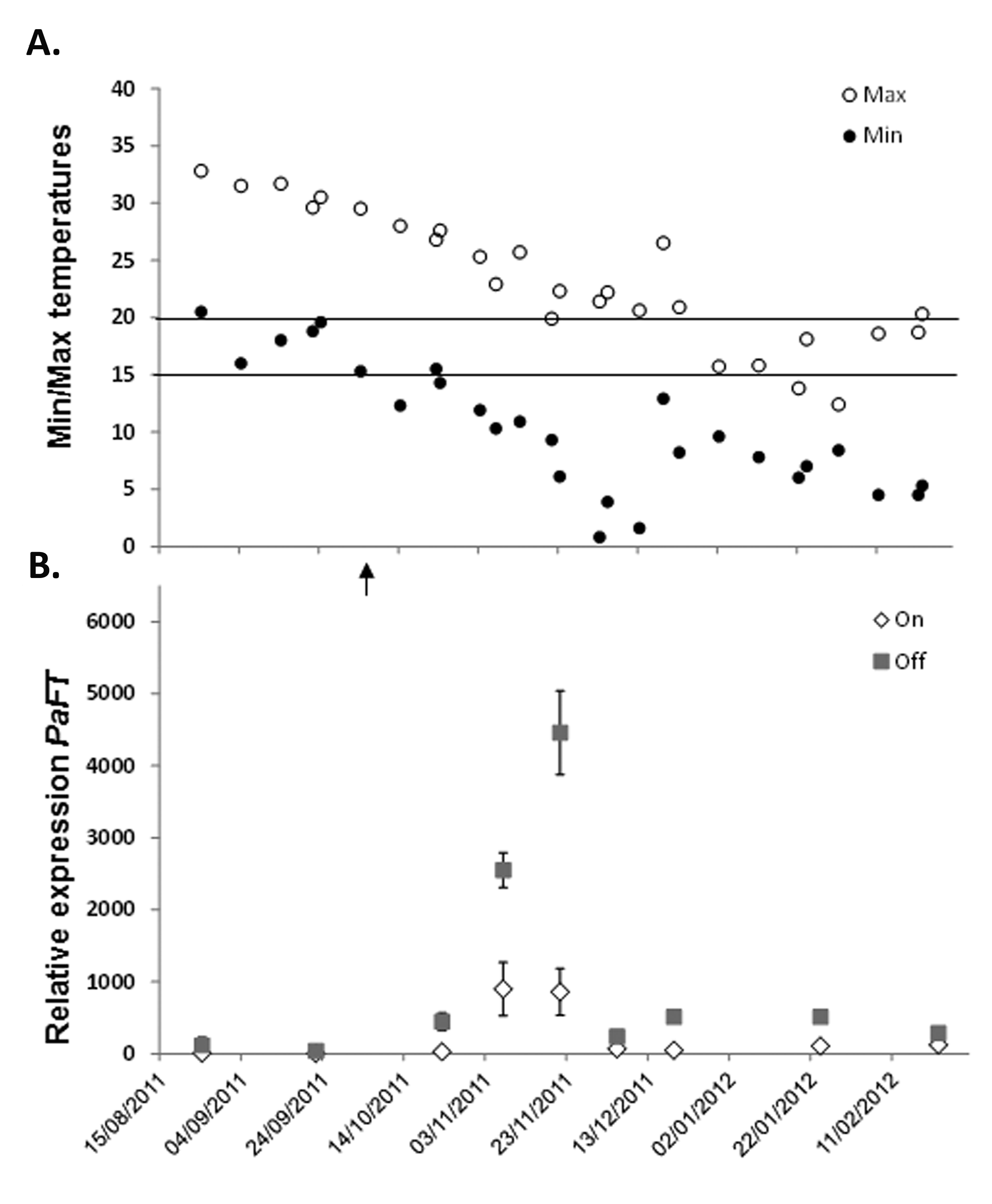

Supplement: Figure S3 — The increase in PaFT mRNA levels correlates with a decrease in temperature levels. Minimal and maximal temperatures recorded from mid-August until the beginning of March at the Tel Mond stiation near kibbutz Eyal, where experimental ‘Hass' trees were grown (A). The arrow denotes the initial date when the minimal temperature dropped below 15°C. Seasonal expression profiles of PaFT in leaves from fully loaded (on) and completely de-fruited (off) ‘Hass' trees are shown. Off trees correspond to trees from which fruit load was completely removed in July (B). Data are means±SE of three independent replicates (n = 3 trees). (TIF) [file pone.0110613.s003.tif]
